# China’s COVID-19 response for the protection of rural communities

**DOI:** 10.1371/journal.pntd.0009018

**Published:** 2021-02-02

**Authors:** Yaxin Zhu, Shenglin Zhao, Bo Qu

**Affiliations:** School of Public Health, China Medical University, Shenyang, Liaoning Province, P.R. China; University of Queensland School of Veterinary Science, AUSTRALIA

In response to the outbreak of the Coronavirus Disease 2019 (COVID-19), the Chinese government has initiated a rapid response. Almost all of China’s provinces, municipalities, and autonomous regions have launched a Level 1 response to this public health emergency since January 26, 2020. In China, a large number of residents live in rural communities (40.8% of the total population, ranking second in the world after India), the health of whom is closely linked to China’s development. Therefore, national-level strategies targeted for rural areas have been issued along with community-based outbreak response tactics, which have been implemented across China since January 30, 2020 [1,2]. In response to China’s epidemic, region-specific and tiered measures for COVID-19 control have been adopted by localities at different risk levels since February 28, 2020 [3]. This period was a crucial stage to stem the spread, and initial progress in containing the virus has been made. Thus, this viewpoint summarizes key national-level COVID-19 epidemic response strategies, specifically targeted for China’s rural areas during this period, which may be useful for rural communities of other countries.

China’s government has emphasized the importance and urgency of COVID-19 prevention in rural areas [1]. The outbreak happened before the Lunar New Year holiday, when millions of rural migrant workers return to their respective homes via public transportation (e.g., by planes, trains, and busses), which heightened the risk of transmissions. Considering the timing of the outbreak and the inadequate sanitation applied throughout rural communities, COVID-19 prevention in China’s rural areas has become a priority. Mass gatherings were banned, including all New Year celebrations, and traffic connecting rural communities was restricted. Rural communities were empowered to prevent and control the epidemic. Both material and human resources have been channeled down to the community level with the aim to strengthen the capacity for prevention and control of COVID-19 in rural communities.

Rural healthcare organizations and relevant personnel have played a great role in preventing the spread of COVID-19. Medical protective equipment and health personnel have been provided by the government to ensure sufficient supply for rural healthcare organizations. These organizations have taken the responsibility to cooperate with higher health authorities and conduct epidemic monitoring, medical screening, follow-up contacts, preexamination triage, and referrals. All information had to be reported daily. The personnel had been trained according to the national technical guidelines [4], with the aim to improve their competence toward preventing and controlling the spread of COVID-19, via measures such as identification, preliminary treatment, environmental disinfection, and protection.

Strengthened prevention measures have targeted the most at-risk population, including returnees from high-risk regions, such as Hubei Province, and people who had been in contact with identified COVID-19 cases. They were either asked to quarantine themselves at home or were taken to special quarantine facilities, where they could be monitored for the onset of symptoms. The health status (e.g., body temperature and symptoms) of all residents has been closely monitored and recorded, with the aim to stop the concealment or underreporting of infections.

Since rural communities are located in remote areas where residents live in a dispersed manner, information technology has been widely applied for COVID-19 prevention among rural communities, with the aim to apply the “Internet Plus” and thus enhance COVID-19 prevention [5]. To prevent panic and properly manage relevant information, the knowledge about COVID-19 and personal protection measures [6] have been made widely available by a massive routine media campaign such as through digital information platforms, community radio, propaganda material, and prevention slogans. The means with which information was transmitted digitally enjoyed great popularity among rural residents and provided fast and efficient transmission of preventive information.

For example, “WeCounty” was an important digital information platform for COVID-19 prevention among rural areas and covered 15,000 rural communities across China [5]. During the outbreak response, the “WeCounty” platform released and disseminated thousands of COVID-19 epidemic information posts and public protection guidelines authorized by the government, which have been accessed by over 590,000 rural residents. This platform also played an important role in monitoring public sentiment. In addition, this platform also cooperated with other internet medical consulting platforms and offered a free online medical consulting service. More than 540,000 rural residents participated in this service, which effectively reduced the risk of COVID-19 cross infection.

Moreover, drones have been deployed for the detection of fever, the monitoring of crowd activity, and environmental disinfection to boost COVID-19 prevention in rural areas, as they offer higher propagation speed, larger coverage, and do not require physical contact [7]. The administrative departments of receiving communities were responsible for managing the use of drone technology. Drones worked as an “air broadcasting” service to disseminate COVID-19 prevention information to rural residents at regular intervals. Spraying drones have also been applied to efficiently disinfect public places. In addition, to allow the frontline disease control staff to collect body temperature data for fever detection without physical contact, drones equipped with a thermometer were used for measuring body temperatures in some rural communities. COVID-19 control staff operated the drones remotely, using real-time recording and broadcast functionality to monitor gatherings and mask adoption in public places. Using the information collected by the drones, the staff could immediately disperse such crowds and ask people to put on their masks. The use of the drones has considerably improved the monitoring efficiency.

In general, the whole nation has been united to participate with high compliance in the “strengthen society-wide efforts to prevent and control the epidemic” program. However, a few adverse events still happened due to some people not complying with COVID-19 preventive measures [8]. Communities would provide support and conduct persuasion, orientation, or warning to the individuals not complying with preventive measures. If these measures still did not work, the criminal law was applied to maintain public health security and ensure the success of COVID-19 prevention. To strengthen the legal safeguards for epidemic prevention and control, China’s Supreme People’s Court, Supreme People’s Procuratorate, Ministry of Public Security, and Ministry of Justice jointly issued relevant COVID-19 prevention policies and took firm action against epidemic-related crimes [8,9]. The policy clarified and defined the criminal characteristic of the actions that do not comply with the national management of COVID-19 and led to serious epidemic spread consequences [9]. Moreover, the government has increased efforts to raise public legal awareness and guide people to act within the parameters of the law [8–10].

Since COVID-19 has become a global pandemic, it has become necessary to share information as quickly as possible and to form strong synergies, so that the maximum effort can be exerted to help decision-makers with formulating strategies to fight this new virus and safeguard global public health [2,11,12]. On March 24, 2020, the National Health Commission of the People’s Republic of China issued a statement that the domestic coronavirus outbreak has been contained in China [13]. In early May, the public health emergency response was updated to Level 2 or lower throughout China [14]. The effective COVID-19 response of China’s rural communities could provide a valuable reference to promote best practices in the prevention and control of COVID-19 in rural areas of other countries. It is noteworthy that these prevention measures should be adapted so that they incorporate the local culture and policy, which may help to translate the successful prevention experience to local settings.
